# ESCRT-III Membrane Trafficking Misregulation Contributes To Fragile X Syndrome Synaptic Defects

**DOI:** 10.1038/s41598-017-09103-6

**Published:** 2017-08-17

**Authors:** Dominic J. Vita, Kendal Broadie

**Affiliations:** 1 Vanderbilt University, Department of Biological Sciences, Nashville, Tennessee 37235 USA; 2 Vanderbilt University, Kennedy Center for Research on Human Development, Nashville, Tennessee 37235 USA; 3 Vanderbilt University, Vanderbilt Brain Institute, Nashville, Tennessee 37235 USA

## Abstract

The leading cause of heritable intellectual disability (ID) and autism spectrum disorders (ASD), Fragile X syndrome (FXS), is caused by loss of the mRNA-binding translational suppressor Fragile X Mental Retardation Protein (FMRP). In the *Drosophila* FXS disease model, we found FMRP binds *shrub* mRNA (human Chmp4) to repress Shrub expression, causing overexpression during the disease state early-use critical period. The FXS hallmark is synaptic overelaboration causing circuit hyperconnectivity. Testing innervation of a central brain learning/memory center, we found FMRP loss and Shrub overexpression similarly increase connectivity. The ESCRT-III core protein Shrub has a central role in endosome-to-multivesicular body membrane trafficking, with synaptic requirements resembling FMRP. Consistently, we found FMRP loss and Shrub overexpression similarly elevate endosomes and result in the arrested accumulation of enlarged intraluminal vesicles within synaptic boutons. Importantly, genetic correction of Shrub levels in the FXS model prevents synaptic membrane trafficking defects and strongly restores innervation. These results reveal a new molecular mechanism underpinning the FXS disease state.

## Introduction

During normal brain maturation, neurons extend excess axonal and dendritic processes in search of appropriate synaptic partners. Superfluous synapses are later selectively eliminated, with less active connections pruned away and more active connections strengthened and stabilized into maturity^1^. Such activity-dependent (A-D) synapse remodeling occurs predominantly during early-use critical periods^2^. This synaptic refinement is necessary to establish proper neural circuit architecture, optimize function and tune behavioral output. Defects in this mechanism are linked to intellectual disability (ID) and autism spectrum disorder (ASD), including the most common inherited form of Fragile X syndrome (FXS)^2–5^. Most FXS cases arise from trinucleotide repeat expansion in the 5′ UTR of the *fragile x mental retardation 1* (*fmr1*) gene, leading to hypermethylation and transcriptional silencing of the Fragile X Mental Retardation Protein (FMRP) gene product^6, 7^. FMRP functions as a selective mRNA-binding protein, predominantly acting to suppress translation^8–11^. Both FXS patients and animal disease models lacking FMRP exhibit an increased number of overelaborated, immature synapses^11–13^. Identifying the molecular translation mechanisms resulting in these connectivity defects is of central importance in combating the disease condition.

The *Drosophila* FXS model has proven invaluable for identifying molecular pathways underpinning synaptic impairments^11, 14–18^. Conserved phenotypes include synaptic overgrowth^17^, loss of A-D synaptic refinement during critical periods^4^, and severe learning/memory deficits^19, 20^. Importantly, impairments are fully rescued through transgenic introduction of human FMRP, proving evolutionarily conserved function^15^. To discover new FMRP targets, we performed a developmental proteomics screen for brain protein changes in the *Drosophila* FXS model^21^. Our most exciting hit was the Endosomal Sorting Complex Required for Transport III (ESCRT-III) core protein Shrub (yeast Snf7/Vps32, human CHMP4)^22–24^. Importantly, recent *Drosophila* studies have shown key roles for the ESCRT pathway in neural mechanisms highly reminiscent of the FXS disease condition^24–28^. In particular, *shrub* mutant neurons display process pruning impairments resulting in an excessive number of branched termini^24, 26–28^, a hallmark phenotype characteristic of the *Drosophila* FXS model^4, 9, 17, 29^. Taken together, these studies suggest a probable link between misregulation of the ESCRT-III core protein Shrub and neural circuit synaptic refinement defects in the FXS disease state.

The ESCRT pathway was defined for its role in endosome-to-multivesicular body (MVB) membrane trafficking^22, 30–32^. It consists of four protein complexes (ESCRT-0,I,II,III) and the associated AAA-ATPase Vps4, together with numerous auxiliary proteins^33^. The ESCRT-III core protein Shrub drives reverse-topology membrane abscission, budding of membrane away from the cytoplasm^34^. Shrub monomers polymerize head-to-tail, creating spiral, helical arrays on membranes causing inward curvature^35–38^. Importantly, unlike the other ESCRTs, ESCRT-III subunits exist as auto-inhibited monomers in the cytosol until recruited to membranes^25, 39–41^. As a result of sequential recruitment, relative protein stoichiometry is absolutely pivotal for proper ESCRT-III function^41–43^. Too much or too little of ESCRT-III components results in similar impaired function and membrane trafficking aberrations, often reported as increased, enlarged endocytic organelles due to blocked endosomal maturation^22, 24, 41, 43^. ESCRT-III also regulates plasma membrane remodeling^44^ and synaptic connectivity^24, 26–28^. These extensive studies suggest a link between misregulation of ESCRT-III core protein Shrub mediated membrane trafficking and synaptic refinement defects in the FXS condition.

In the current study, we test the hypothesis that FMRP negatively regulates ESCRT-III Shrub to control both synaptic membrane trafficking and neural circuit connectivity. Using RNA immunoprecipitation^11, 45^ and brain Western blot assays^9, 10^, we find FMRP binds *shrub* mRNA to negatively regulate Shrub protein levels during a defined early-use critical period immediately following eclosion. Since FMRP functions in an activity sensor mechanism to mediate the A-D translation of mRNA targets^4^, we use optogenetics to reveal that Shrub expression is positively upregulated by neural activity dependent on FMRP. Using well-defined olfactory projection neuron (PN) innervation of the central brain mushroom body (MB) learning/memory center^46, 47^, we find that Shrub gain-of-function (GOF) closely phenocopies FMRP loss-of-function (LOF) synaptic errors with a combination of confocal and transmission electron microscopy (TEM) studies. We further find accumulated endocytic organelles with arrested membrane trafficking intermediates in both Shrub GOF and FMRP LOF synapses, consistent with previous reports of ESCRT-III defects^31, 48–50^. Finally, we strongly mitigate FMRP LOF membrane trafficking and synaptic connectivity defects through reducing Shrub levels in the FXS disease model. Together, these results link ESCRT-III membrane trafficking impairments at the synapse to synaptic connectivity defects as a new causative mechanism in the FXS disease state.

## Results

### FMRP binds shrub mRNA to negatively regulate expression during the A-D critical period

**Figure 1:**
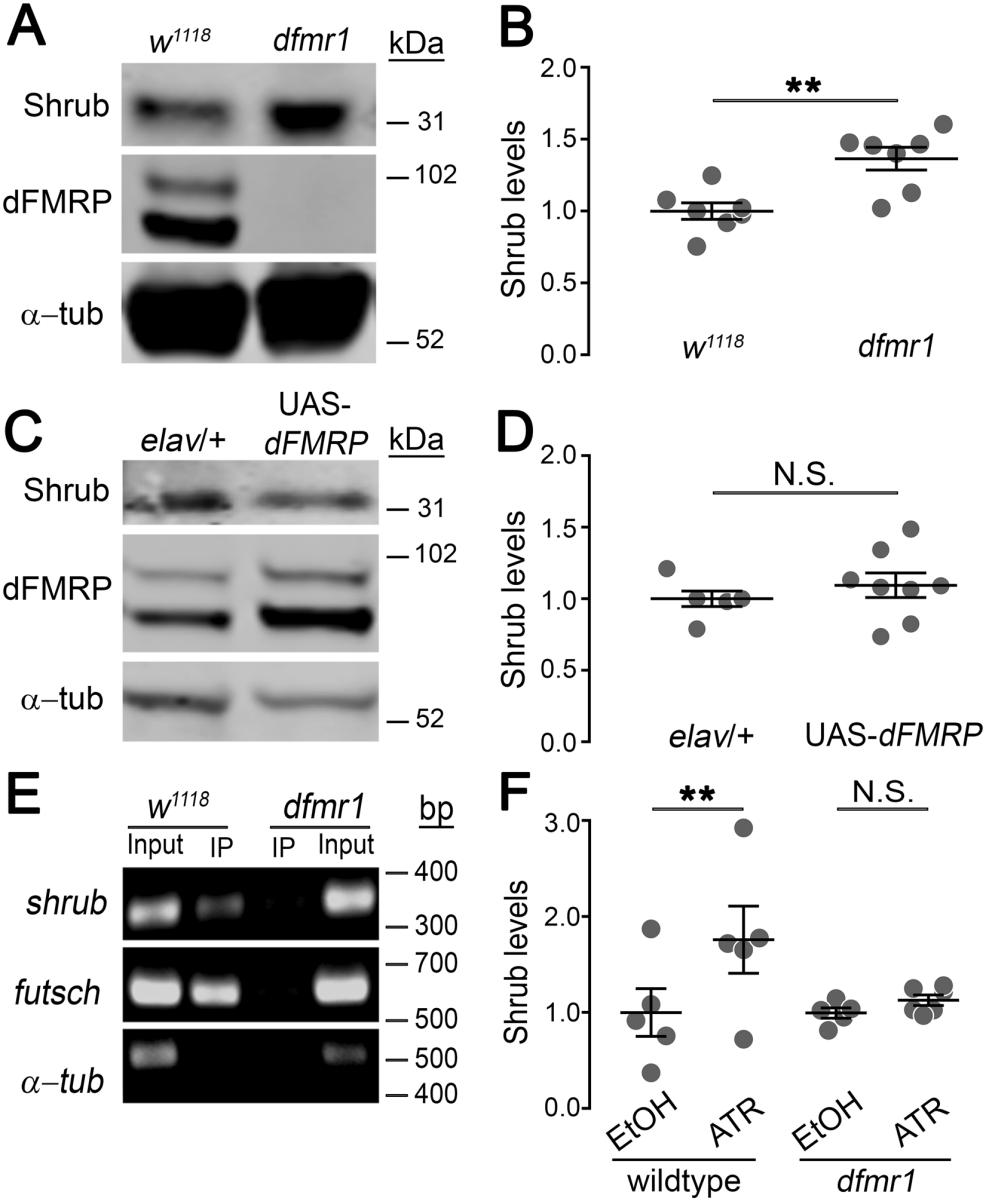
FMRP negatively regulates brain Shrub levels during the A–D critical period. (**A**) Brain Western blot comparing *w* ^1118^ background control to *dfmr1* ^*50M*^ null mutant at 0–3 hr post eclosion (3PE). Proteins probed indicated on left, molecular weights on right. (**B**) Dot plots of the normalized Shrub data points, with mean ± SEM. (**C**) Brain Western blot comparing driver control (*elav*-Gal4/+) to UAS-dFMRP rescue (*elav*-Gal4/+; UAS-*dfmr1* ^*9557–3*^, *dfmr1* ^*50M*^ */dfmr1* ^*50M*^) at 3PE. Proteins probed indicated on left, molecular weights on right. (**D**) Dot plots of normalized Shrub data points, with mean ± SEM. (**E**) RNA immunoprecipitation (RIP) comparing *w* ^1118^ control to *dfmr1* ^*50M*^ null. RNAs probed indicated on left, molecular weights on right. (**F**) Optogenetic control of Shrub in *elav*-Gal4/UAS-ChR2(H134R)-mCherry control animals (left) and *elav*-Gal4/UAS-ChR2(H134R)-mCherry; *dfmr1* ^*50M*^ mutants (right) reared on EtOH vehicle (not stimulated) or ATR (stimulated) food. Animals were illuminated with 5 Hz blue light for 2 hr, with brains tested by anti-Shrub Western blot. Dot plots of normalized Shrub data points, with mean ± SEM. Statistics done with two tailed unpaired t-tests, indicated as **P < 0.01 and N.S. *P* > 0.05.

Based on a developmental brain proteomics screen in the *Drosophila* FXS disease model, we identified a transient change in Shrub protein levels during the early-use critical period^21^. To test this candidate, we performed brain Western blots from staged animals during a defined window (0–3 hours post-eclosion; 3PE), established as the peak for FMRP-/activity-dependent refinement of the MB learning/memory center in the central brain^4, 10^. Using well-characterized antibodies for Shrub^24, 27, 51, 52^ and dFMRP^4, 5, 11, 15^, we find brain Shrub levels to be consistently increased in the *dfmr1* null mutants compared to *w* ^1118^ genetic background controls (Fig. 1A). In quantified comparisons normalized to α-tubulin controls, Shrub levels are elevated ~45% in the FXS disease model brain compared to matched controls (*p* < 0.01; Fig. 1B). To test the specificity of FMRP regulation on Shrub, we expressed wildtype FMRP in neurons under control of the *elav*-Gal4 driver in the *dfmr1* null background (Fig. 1C). This FMRP rescue reduces Shrub protein to levels indistinguishable from controls. In quantified comparisons normalized to α-tubulin controls, Shrub levels are the same as transgenic driver alone controls (*p* > 0.05; Fig. 1D). These results demonstrate a role for FMRP in limiting Shrub expression in neurons specifically during the early-use critical period.

FMRP is an mRNA-binding protein^53–55^, typically exerting regulatory suppression of translation via interaction with select targets^9, 11, 56^. To test if FMRP directly interacts with *shrub* mRNA, we employed RNA immunoprecipitation (RIP) to assay FMRP-RNA binding in *w* ^1118^ controls compared to *dfmr1* nulls (Fig. 1E). In these assays, antibodies conjugated to magnetic beads immunoprecipitate bound mRNAs, which are reverse transcribed and probed using target-specific primers. Brain lysates prepared from newly eclosed animals (3PE) were incubated with specific anti-FMRP antibodies to test for *shrub* mRNA binding (Fig. 1E). As controls, we used a characterized *futsch/map1b* mRNA target of FMRP (positive control) and *α*-*tubulin* mRNA which does not bind FMRP (negative control)^11^. FMRP bound mRNAs were first purified with trizole and then reverse transcribed with RT-PCR. In *w* ^1118^ controls, there is strong enrichment for *shrub* mRNA bound to FMRP in the immunoprecipitation (IP) lanes (Fig. 1E). In contrast, *dfmr1* null mutants lacking FMRP show no detectable *shrub* mRNA binding. These results are mirrored with the *futsch* mRNA positive control, but there is no enrichment for the *α*-*tubulin* mRNA negative control (Fig. 1E). These findings demonstrate FMRP directly interacts with *shrub* mRNA in the brain to suppress Shrub protein translation.

FMRP functions in an activity sensor mechanism to mediate activity-dependent (A-D) changes in protein expression^2, 4, 10^. Upon neural stimulation, FMRP is dephosphorylated leading to release of bound, repressed mRNA targets and driving A-D translational upregulation, including its own transcript^57^. To test the hypothesis of A-D Shrub elevation during the early-use window identified above, we used optogenetic manipulation to elevate neural activity (Fig. 1F). Staged animals expressing light-activated channelrhodopsin (UAS-ChR2(H134R)-mCherry) driven with *elav*-Gal4 were raised on food containing either 1) the essential cofactor all-trans retinal (ATR; experimental) or 2) ethanol vehicle only (EtOH; controls). Both sets of animals were then exposed to 5 Hz blue light pulses for the 2 hours immediately following eclosion. As above, brains were then dissected and probed with anti-Shrub and -dFMRP (Fig. 1F). Compared to vehicle controls (EtOH), stimulated animals reared on ATR show a >50% increase in brain Shrub levels (*p* < 0.01; Fig. 1F, left). There is also a slight but significant increase in FMRP levels (*p* < 0.05). In *dfmr1* null mutants, there is no change in brain Shrub levels with optogenetic stimulation (Fig. 1F, right), indicating that FMRP is absolutely required to regulate Shrub expression in an A-D mechanism during the early-use critical period.

As the next step, we proceeded to test the contribution of elevated Shrub expression to the primary synaptic connectivity hallmark phenotypes characterizing the FXS disease state. In subsequent studies, Shrub is over-expressed in target neurons to generate a gain-of-function (GOF) condition mimicking the *dfmr1* null phenotype (Fig. 1A,B). Brain Western blots at 3PE show UAS-*shrub* driven by *elav*-Gal4 elevates Shrub protein levels in neurons compared to the transgenic control (*elav*-Gal4/+), with no change in dFMRP protein levels (Fig. S1A). Quantified measurements show a significant increase in brain Shrub levels, comparable to the elevation characterizing the FXS disease model (Fig. S1B). To correct elevated Shrub levels, we use a *shrub* ^*4*^ null heterozygous (*shrub* ^*4*^/+) loss-of-function (LOF) condition^24^. Brain Western blots show this mutant in the *dfmr1* background (*shrub* ^*4*^/+; *dfmr1*) significantly reduces brain Shrub levels compared to *dfmr1* alone (Fig. S1C,D). Importantly, introducing *shrub* ^*4*^/+ into animals lacking FMRP (*shrub* ^*4*^/+; *dfmr1*.) completely restores normal brain Shrub levels (Fig. S1E). Quantified measurements show no significant difference in brain Shrub levels in the rescued mutant animals compared to genetic background controls (*p* > 0.05; Fig. S1F). These Shrub manipulations will be compared to the FXS disease model in all subsequent experiments.

### Shrub GOF phenocopies FMRP LOF synaptic overelaboration

**Figure 2:**
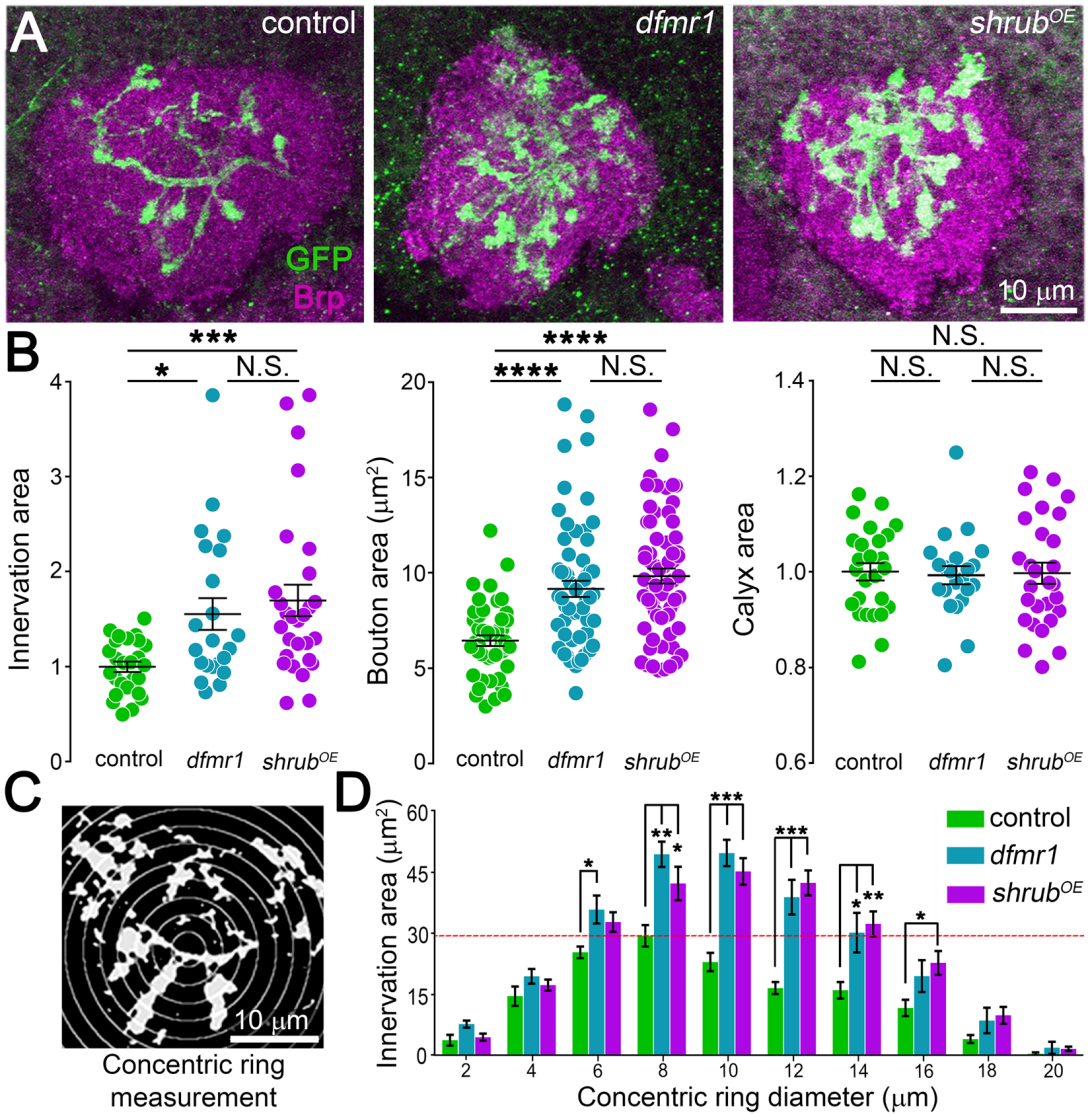
FMRP LOF and Shrub GOF similarly increase projection neuron innervation. (**A**) Representative confocal images of central brain mushroom body calyx in transgenic control (UAS-mCD8::GFP/+; Nrv3-Gal4/+), the *dfmr1* null mutant (UAS-mCD8::GFP/+; Nrv3-Gal4, *dfmr1* ^*50M*^/*dfmr1* ^*50M*^) and *shrub* over-expression (*shrub* ^*OE*^) GOF line (UAS-mCD8::GFP/+; Nrv3-Gal4/UAS-Shrub). PN axons (green) in calyx (Brp labeled, magenta) at 3PE. (**B**) Quantification of mushroom body calyx innervation area (left), single projection neuron bouton area (middle) and total calyx area (right) in dot plots showing all data points with mean ± SEM indicated for all three genotypes. (**C**) Sholl analysis of PN innervation of the mushroom body calyx based on centered 2 μm concentric rings. (**D**) Quantification of innervation area per ring for the above three genotypes. Red dotted line indicates maximum control innervation. Statistics done with one way ANOVA with the Kruskal-Wallis test followed by a Dunn’s multiple comparison test post hoc indicated as *P* > 0.05 (N.S.), *P < 0.05, **P < 0.01, ***P < 0.001 and ****P < 0.0001.

FMRP-deficient neurons display overelaborated synaptic connectivity in FXS patients and animal models^12, 13, 58^. To test the role of Shrub elevation on this hallmark phenotype, we assayed well-characterized olfactory projection neuron (PN) innervation of the central brain mushroom body (MB) calyx learning and memory center^46, 47^. We recently demonstrated PNs manifest FMRP- and activity-dependent critical period connectivity^4, 59^. Using a new, highly-restricted Gal4 (Nrv3-Gal4)^60^ to drive a membrane marker (UAS-mCD8-GFP) in a small PN subset, MB calyx innervation was cross-compared between *dfmr1* nulls and Shrub over-expression (*shrub* ^*OE*^) in targeted neurons (Fig. 2). In transgenic controls (Nrv3-Gal4/+), there is a consistent pattern of MB calyx microglomeruli innervation (Fig. 2A, left). In sharp contrast, both *dfmr1* LOF (middle) and targeted *shrub* GOF (right) cause increased PN innervation, with similar overelaborated and overextended MB calyx areas (Fig. 2A). In quantified comparisons, *dfmr1* (1.55 ± 0.17, *n* = 22 hemispheres, *p* < 0.05) and *shrub* ^*OE*^ (1.7 ± 0.17, *n* = 28, *p* < 0.001) have significantly increased PN innervation areas normalized to control (1.0 ± 0.06, *n* = 26), but no significant difference (*p* > 0.05) with each other (Fig. 2B, left). Individual PN synaptic boutons also show similarly enlarged areas in both *dfmr1* (9.17 ± 0.43 μm^2^, *n* = 61 boutons, *p* < 0.0001) and *shrub* ^*OE*^ (9.84 ± 0.38 μm^2^, *n* = 70, *p* < 0.0001) compared to controls (6.45 ± 0.43 μm^2^, *n* = 47). Once again, PN-targeted *shrub* ^*OE*^ phenocopies the FXS disease model (*p* > 0.05; Fig. 2B, middle). Importantly, MB calyx area is indistinguishable between genotypes, showing mutant defects map specifically to increased PN innervation (Fig. 2B, right).

In examining innervation patterns, it is apparent that *dfmr1* null and *shrub* ^*OE*^ PNs extend into MB calyx regions not innervated in controls (Fig. 2A). Similar connectivity defects have been reported previously in the *Drosophila* FXS disease model, for example in central brain circadian clock neurons^5^. To test the role of Shrub elevation on this *dfmr1* phenotype, we did Sholl analyses on the spatial extent of MB calyx innervation (Fig. 2C). A series of 2 μm concentric rings centered on the calyx centroid was superimposed on each Nrv3-Gal4 > UAS-mCD8-GFP image, with GFP-positive areas from each ring quantified (Fig. 2C). Compared to transgenic controls (Nrv3-Gal4/+), both *dfmr1* and *shrub* ^*OE*^ exhibit similar striking expansion of innervation areas, which reach far further from the MB calyx centroid (Fig. 2D). In controls, innervation area peaks around 30 μm^2^ at 8 μm from the MB center (dotted red line). In contrast, both *dfmr1* null and PN-targeted *shrub* ^*OE*^ exhibit similar higher levels of innervation (40–50 μm^2^) for further distances from the calyx centroid (8–12 μm; Fig. 2D). Innervation areas surpassed controls in *dfmr1* LOF and *shrub* ^*OE*^ GOF in all rings between 6 and 16 μm, with the largest differences at 8 μm (*dfmr1*, *p* < 0.01; *shrub* ^*OE*^, *p* < 0.05), 10 μm (*dfmr1*, *p* < 0.001; *shrub* ^*OE*^, *p* < 0.001), 12 μm (*dfmr1*, *p* < 0.001; *shrub* ^*OE*^, *p* < 0.001) and 14 μm (*dfmr1*, *p* < 0.05; *shrub* ^*OE*^, *p* < 0.01). Importantly, there is no significant difference between *dfmr1* and *shrub* ^*OE*^ in any ring (Fig. 2D). Taken together, these results show PN-targeted *shrub* ^*OE*^ phenocopies FXS model overelaboration of MB calyx innervation, including increased area, enlarged synaptic boutons and expanded spatial ramification. These results suggest elevated Shrub levels drive FXS synaptic connectivity errors. Since the mechanistic consequences of Shrub GOF are unknown, we next examined Shrub function in PN synaptic terminals.

### Shrub GOF phenocopies FMRP LOF synaptic membrane trafficking defects

**Figure 3:**
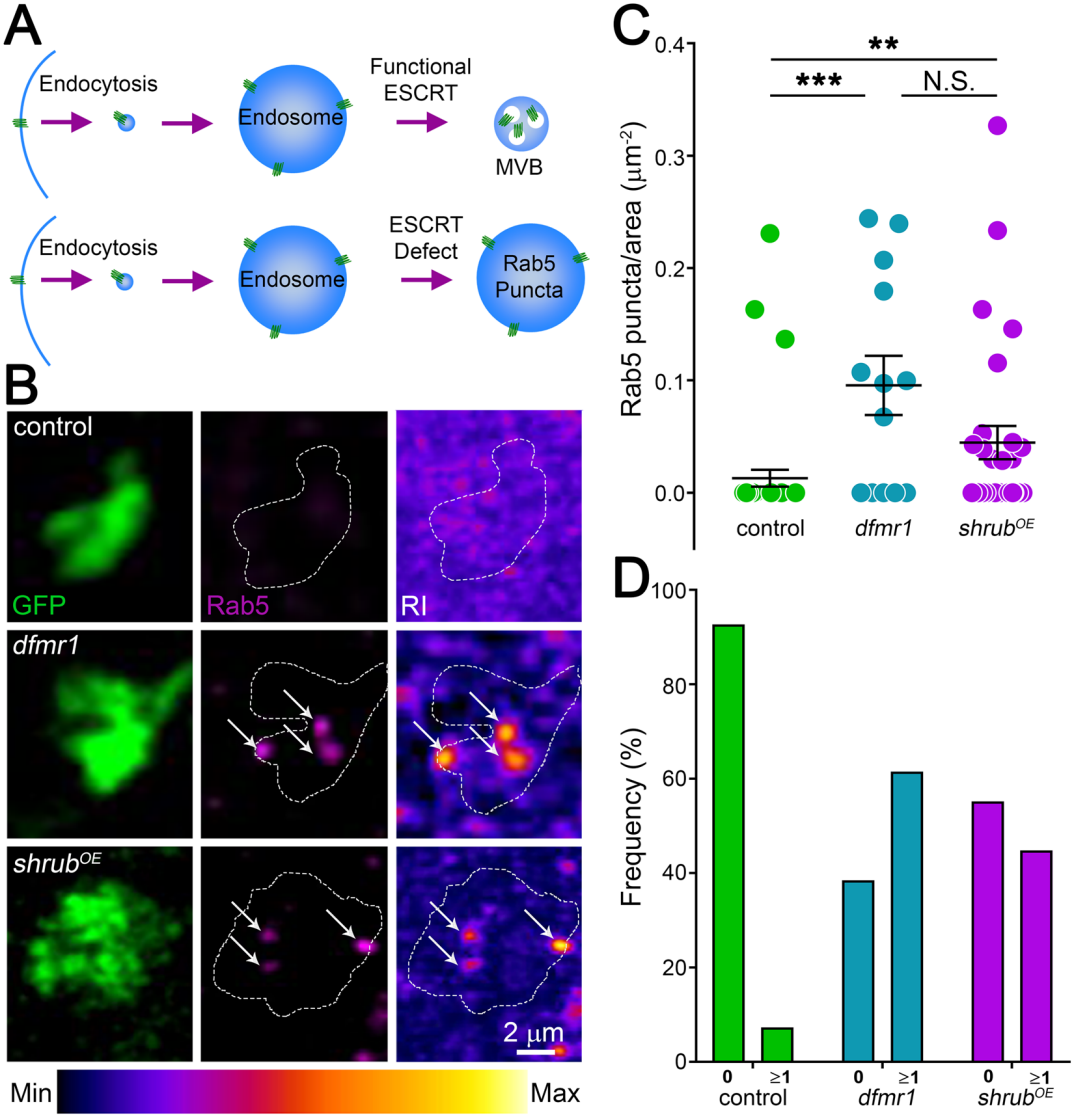
FMRP LOF and Shrub GOF similarly increase PN synaptic endosomes. (**A**) Schematic of ESCRT trafficking of Rab5-positive endosomes. Top: Normally functioning ESCRT in which endocytosis leads to endosomes and subsequent multivesicular body (MVB) formation. Bottom: Defective ESCRT in which trafficking is arrested leading to Rab5-positive enlarged endosomes. (**B**) Representative confocal images of single PN synaptic boutons (green) labeled with anti-Rab5 (magenta) comparing the transgenic control (top: UAS-mCD8::GFP/+; Nrv3-Gal4/+), *dfmr1* null mutant (middle: UAS-mCD8::GFP/+; Nrv3-Gal4, *dfmr1* ^*50M*^/*dfmr1* ^*50M*^) and *shrub* ^*OE*^ (bottom: UAS-mCD8::GFP/+; Nrv3-Gal4/UAS-Shrub). The right column shows heat maps of Rab5 labeling, with the range indicated below. (**C**) Quantification dot plots showing data points with mean ± SEM of the above three genotypes displaying Rab5 puncta per synaptic bouton area. (**D**) Quantification of the bouton frequency with 0 or ≥1 Rab5-positive punctae. Statistics done with one way ANOVA with the Kruskal-Wallis test followed by a Dunn’s multiple comparison test post hoc indicated as N.S. *P* > 0.05, **P < 0.01 and ***P < 0.001.

The ESCRT-III core component Shrub (yeast Snf7/Vps32, human CHMP4) has conserved functions in the membrane trafficking^22, 23, 51^. Disruptions in this mechanism result in aberrant, enlarged endocytic organelles^24^ with associated neurological defects^26–28^. Importantly, Shrub LOF and GOF similarly disrupt membrane trafficking^41^, suggesting Shrub elevation in the FXS disease state could impair membrane trafficking to cause synaptic connectivity defects. The small GTPase Rab5 is commonly used to label endosomes^24, 28, 50^, and often presents as aberrant, enlarged punctae in *shrub* mutants (Fig. 3A). We used this marker to test how *dfmr1* LOF and *shrub* ^*OE*^ GOF might affect endosomes in PN synaptic terminals. During normal membrane trafficking, internalized vesicles fuse into endosomes that are subsequently acted upon by ESCRTs to form a multivesicular body (MVB; Fig. 3A, top); however, ESCRT defects result in impaired maturation (bottom). We hypothesized Shrub GOF and FMRP LOF similarly disrupt progression through this pathway to cause an increase in enlarged Rab5-positive endosomes. To test this hypothesis, dissected brains from newly-eclosed animals (3PE) with GFP-marked PNs (Nrv3-Gal4 > UAS-mCD8-GFP) were labeled for Rab5 (Fig. 3B). Single optic slices (<0.5 μm) of PN synaptic boutons were assayed for endosomes. Compared to transgenic controls (Nrv3-Gal4/+), both *dfmr1* nulls and PN-targeted *shrub* ^*OE*^ show a striking increase in Rab5-positive endosome number in PN boutons within the calyx (Fig. 3B). This suggests a similar endocytic membrane trafficking defect.

Using the Rab5 endosomal marker in quantified comparisons, both *dfmr1* null mutants (0.1 ± 0.03 μm^2^, *n* = 23 boutons, *p* < 0.001) and PN-targeted *shrub* ^*OE*^ GOF (0.04 ± 0.01 μm^2^, *n* = 29, *p* < 0.01) show highly significant increases in the number of endosomes per synaptic bouton area compared to controls (Fig. 3C). Importantly, FMRP LOF and Shrub GOF show no significant difference (*p* > 0.05), and are indistinguishable from each other (Fig. 3C). A number of studies report an increase in Rab5-positive endosomal size resulting from ESCRT defects^24, 27, 28^. It should be noted that Rab5-positive endosomes in control PN boutons are typically too small to be resolved with light microscopy, and only become readily detectable when aberrantly enlarged in *dfmr1* LOF and *shrub* GOF conditions (Fig. 3B). Comparing the frequency of detectable Rab5-positive synaptic endosomes across genotypes, <5% of control synaptic boutons contain any measurable endosomes, whereas >50% of both *dfmr1* null and *shrub* ^*OE*^ boutons have large endosomes (Fig. 3D). These findings suggest that small, dynamic endosomes predominate in control boutons, but that more aberrantly enlarged endosomes characterize both *dfmr1* LOF and *shrub* GOF boutons (Fig. 3D), consistent with a local membrane trafficking defect within the synapse. However, these synaptic organelles are at the edge of light microscopy resolution, particularly in control brains, so we next turned to transmission electron microscopy for a more detailed examination of PN synapse structure and membrane trafficking changes due to Shrub elevation in the FXS disease state.

### Shrub GOF phenocopies FMRP LOF synaptic ultrastructural deficits

**Figure 4:**
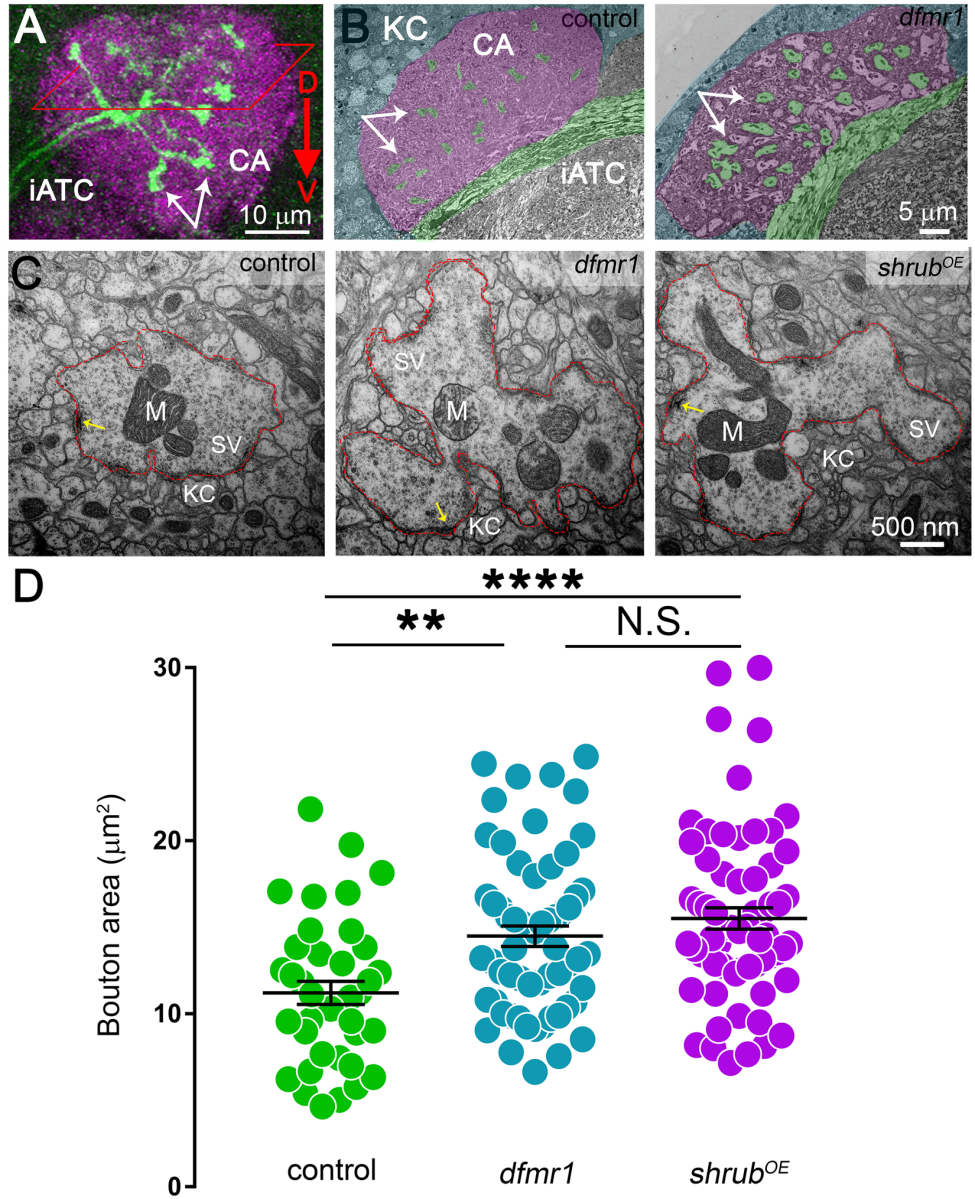
FMRP LOF and Shrub GOF similarly alter PN synaptic ultrastructure. (**A**) Confocal image stack of the mushroom body calyx showing sectioning strategy; white arrows (PN boutons), rhombus (sectional plane) and red arrow (sectional direction). PNs are labeled with UAS-mCD8::GFP/+; Nrv3-Gal4/+ (green) in the MB calyx (magenta). Abbreviations: inner antennocerebral tract (iACT), calyx (CA), dorsal (D) and ventral (V). (**B**) Low magnification (1450X) transmission electron micrographs of *w* ^1118^ genetic background control (left) and *dfmr1* ^*50M*^ null mutant (right) during 3PE. Both images are pseudo-colored as in (**A**): iACT axons and PN boutons (green), calyx (magenta), and Kenyon cells (KC, blue). (**C**) Representative PN synaptic bouton electron micrographs comparing transgenic control (*elav*-Gal4/+), *dfmr1* null (*elav*-Gal4/+; *dfmr1* ^*50M*^) and *shrub* over-expression (*shrub* ^*OE*^) GOF (*elav*-Gal4/+; UAS-Shrub/+). The dotted red lines depict the PN bouton borders; active zone t-bars are shown by yellow arrows, synaptic vesicles pools (SV), mitochondria (M), Kenyon cell (KC). (**D**) Dot plot showing PN bouton area quantification for the above three genotypes, with mean ± SEM. Statistics done with one way ANOVA with the Kruskal-Wallis test followed by a Dunn’s multiple comparison test post hoc indicated as N.S. *P* > 0.05, **P < 0.01 and ****P < 0.0001.

Our lab and others have characterized ultrastructure abnormalities in FMRP-deficient synaptic boutons^17, 61, 62^, albeit never with a focus on PN innervation of the MB calyx. To determine whether increased Shrub levels might affect synaptic ultrastructure in the FXS disease state, we used transmission electron microscopy (TEM) to compare *dfmr1* null and *shrub* ^*OE*^ PN synaptic boutons (Fig. 4). The MB calyx (CA) is located in the posteriodorsal brain region, revealed in light microscopy by dense labeling of the presynaptic marker bruchpilot (purple), with PN axons (green) entering through the inner antennocerebral tract (iACT; Fig. 4A). PN synaptic boutons are easily identified as the largest varicosities (arrows) in the calyx. For TEM studies, acutely dissected brains (3PE) embedded in epoxy resin were oriented with the microtome sectional plane progressing from dorsal to ventral (Fig. 4A, rhombus). Electron micrographs reveal the MB calyx as an oval, fan-shaped nucleus 40–50 μm in cross-sectional diameter (Fig. 4B; pseudo-colored purple), encapsulated on three sides by Kenyon cell (KC) somata (blue). Just anterior to the MB calyx, the iATC is an easily identifiable TEM landmark with longitudinal projections bending into the base of the MB calyx (Fig. 4B; pseudo-colored green). PN synaptic boutons can be seen extending off the iACT into the calyx (green varicosities). Comparing the *dfmr1* null mutant to matched control, there are no detectable differences in the gross MB calyx architecture, including iATC placement, KC cell bodies and calyx size (Fig. 4B).

Comparison of low magnification TEM images of *dfmr1* nulls and controls shows a striking difference in PN boutons in the MB calyx (Fig. 4B). In controls, synaptic boutons are difficult to discriminate at low magnification, whereas *dfmr1* mutants display obvious, enlarged boutons, consistent with published FXS reports^17^. To test whether Shrub GOF has similar effects, we examined *shrub* ^*OE*^ PN boutons in parallel at high magnification (Fig. 4C). Synaptic boutons were defined as large varicosities (red dotted line) completely surrounded by the smaller Kenyon cell (KC) dendritic profiles to form the MB calyx microglomeruli. PN boutons in controls and both mutant conditions contain stereotypical synaptic architecture, including clearly defined synaptic vesicle (SV) pools, multiple mitochondria (M) and active zones with electron-dense t-bars (yellow arrows). There are no apparent differences in any of these parameters (Fig. 4C). However, control boutons are much smaller compared to both *dfmr1* LOF nulls and *shrub* ^*OE*^ GOF animals (Fig. 4C). Quantitative analyses show *dfmr1* null (14.49 ± 0.59 μm^2^, *n* = 61 boutons, *p* < 0.01) and *shrub* ^*OE*^ (15.51 ± 0.62 μm^2^, *n* = 65, *p* < 0.0001) boutons both exhibit significantly increased areas compared to controls (11.21 ± 0.07 μm^2^, *n* = 39; Fig. 4D). Importantly, there is no significant difference between *dfmr1* and *shrub* ^*OE*^ (*p* > 0.05), with both showing an indistinguishable synaptic bouton enlargement (Fig. 4C,D). These results are consistent with increased Shrub levels driving FXS synaptic ultrastructure errors, and suggest membrane trafficking impairment within the synaptic termini as the causative mechanism.

### Shrub GOF phenocopies FMRP LOF synaptic membrane trafficking errors

**Figure 5:**
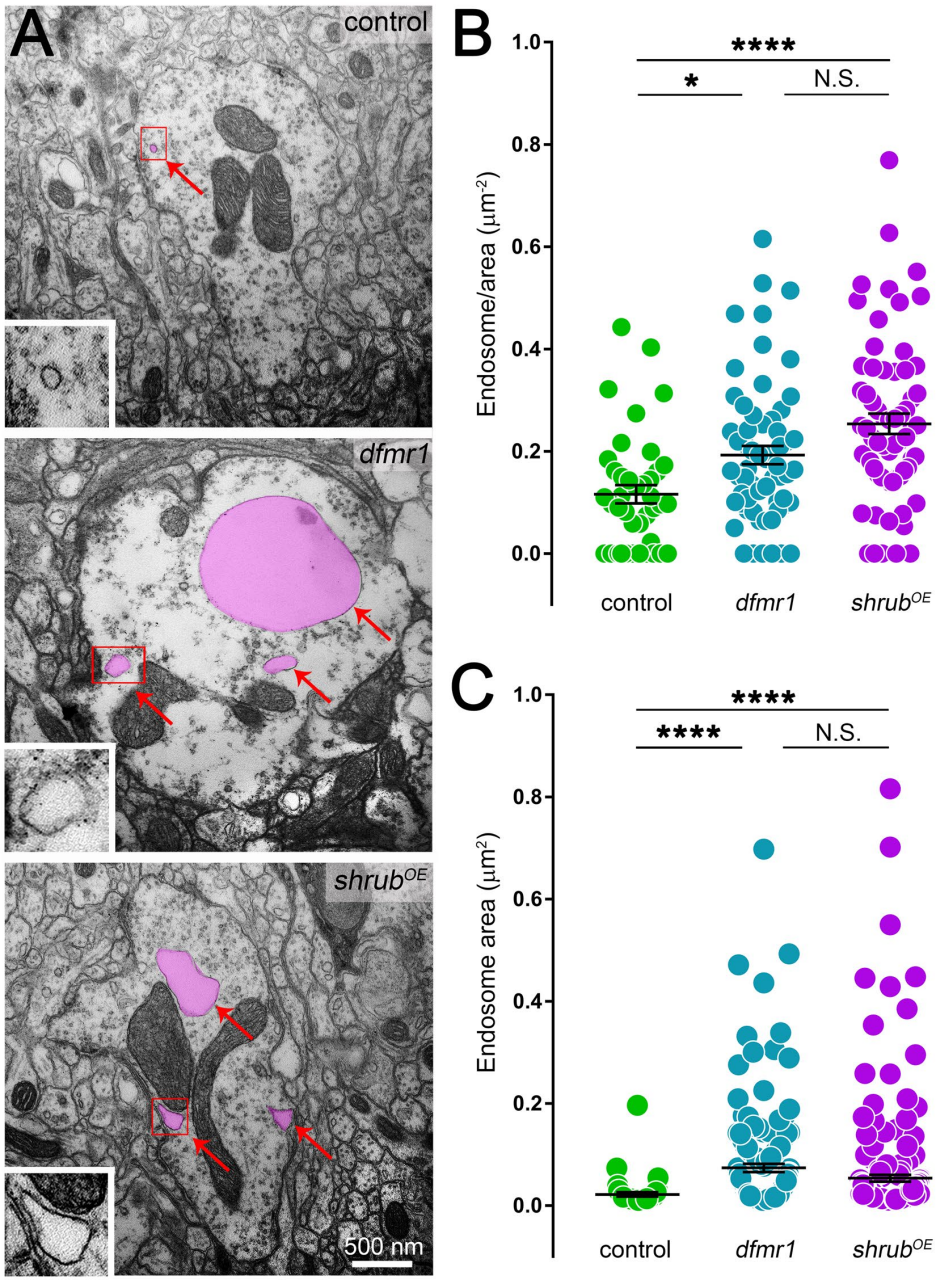
FMRP LOF and Shrub GOF similarly increase PN synaptic endosomic vacuoles. (**A**) Representative electron micrographs of projection neuron synaptic boutons in the MB calyx comparing transgenic control (*elav*-Gal4/+), *dfmr1* null mutant (*elav*-Gal4/+; *dfmr1* ^*50M*^) and *shrub* over-expression (*shrub* ^*OE*^) GOF (*elav*-Gal4/+; UAS-Shrub/+). Typical endosomic vacuole examples are pseudo-colored (magenta), with insets showing enlarged organelle (red box in main image). Dot plot quantification of endosomes per bouton area (**B**) and endosome area (**C**) for all three genotypes, comparing mean ± SEM. Statistics done with one way ANOVA with the Kruskal-Wallis test followed by a Dunn’s multiple comparison test post hoc indicated as N.S. *P* > 0.05, *P < 0.05 and ****P < 0.0001.

To more precisely investigate defects in synaptic membrane trafficking, TEM was next used to analyze ultrastructural changes in PN synaptic bouton organelles. We hypothesized that FMRP LOF and Shrub GOF would cause enlarged, trafficking-arrested endosomes, consistent with Shrub requirements in other contexts^22, 24^. For TEM, we defined endosomes as vacuole organelles with a single membrane and 0.01 μm^2^ minimum area. Such synaptic organelles are present in all genotypes analyzed (Fig. 5A; pseudo-colored purple and insets), with control boutons displaying a low density that never exceeds 0.25 μm^2^ in area (top). In stark contrast, *dfmr1* null and *shrub* ^*OE*^ boutons both present increased numbers of these organelles, with greatly inflated areas (Fig. 5A; middle and bottom). Quantification reveals significantly increased numbers per bouton in *dfmr1* null (0.19 ± 0.02, *n* = 61 boutons, *p* < 0.05) and *shrub* ^*OE*^ (0.25 ± 0.02, *n* = 65, *p* < 0.0001) compared to controls (0.12 ± 0.02, *n* = 40; Fig. 5B). Importantly, there is no significant difference (*p* > 0.05) between FMRP LOF and Shrub GOF boutons (Fig. 5B). In the size of these compartments, there is highly significantly increased areas in both *dfmr1* (0.07 ± 0.008 μm^2^, *n* = 156 endosomes, *p* < 0.0001) and *shrub* ^*OE*^ (0.05 ± 0.007 μm^2^, *n* = 237, *p* < 0.0001) compared to controls (0.02 ± 0.004 μm^2^, *n* = 46; Fig. 5C). Once again, there is no significant difference (*p* > 0.05) between FMRP LOF and Shrub GOF boutons (Fig. 5C). These results are consistent with an ESCRT-III defect and suggest elevated Shrub in the FXS disease state disrupts endocytic membrane trafficking within synaptic terminals.

**Figure 6:**
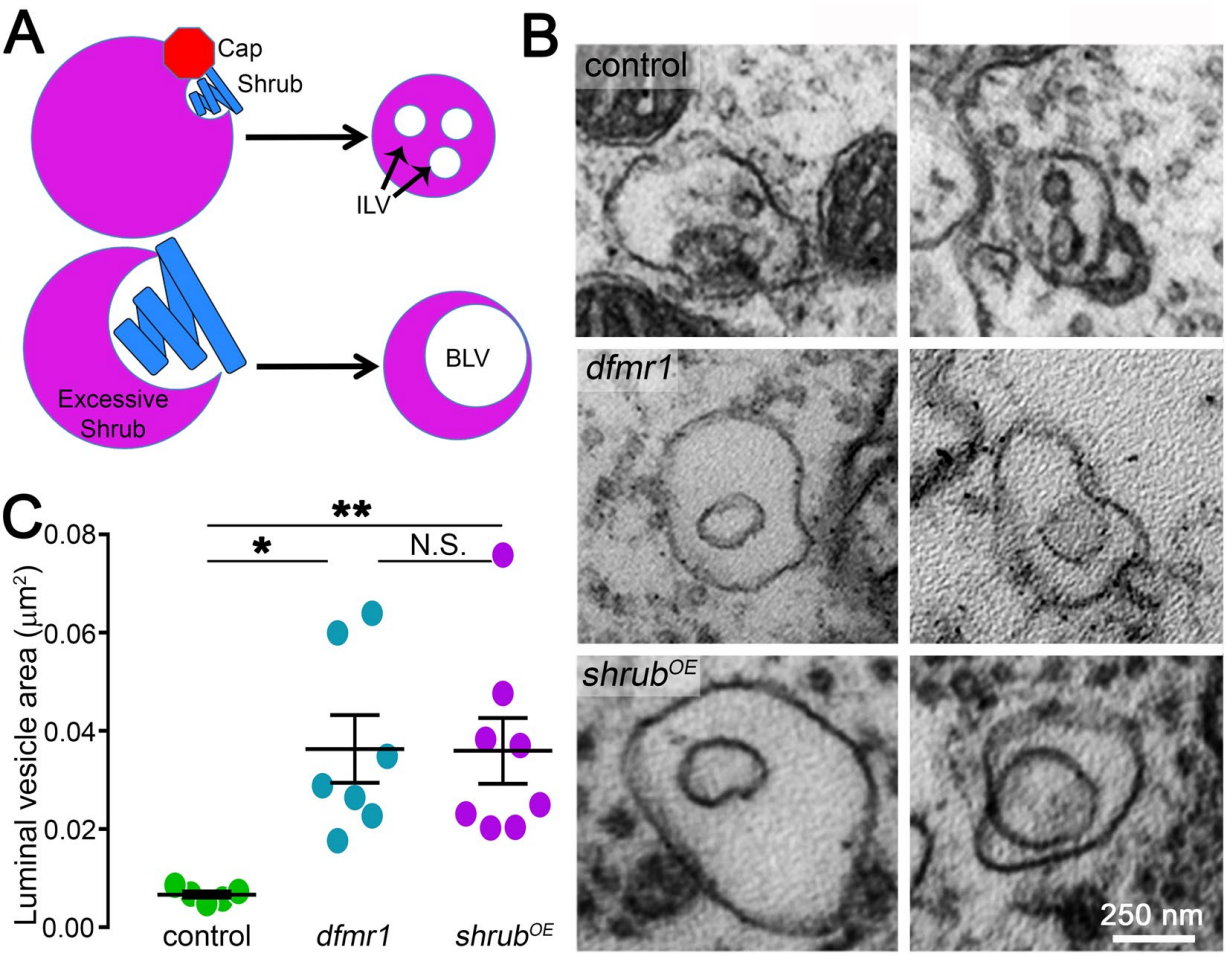
FMRP LOF and Shrub GOF similarly arrest ILV formation in synaptic boutons. (**A**) Schematic depicting how increased Shrub levels impact ILV formation. Top: Proper Shrub levels form spiral protein filaments (blue) that are capped by Vps24 (red octagon) leading to small interluminal vesicle (ILV) formation within MVBs. Bottom: Excessive Shrub levels result in unrestrained filaments (blue), not correctly capped, leading to big luminal vesicle (BLV) formation. (**B**) Representative electron micrographs comparing ILV organelles in transgenic controls (*elav*-Gal4/+) to enlarged BLV organelles characterizing both the *dfmr1* null mutant (*elav*-Gal4/+; *dfmr1* ^*50M*^) and *shrub* over-expression (*shrub* ^*OE*^) GPF (*elav*-Gal4/+; UAS-Shrub/+). (**C**) Quantification dot plot of luminal vesicle areas in all three genotypes, showing mean ± SEM. Statistics done with one way ANOVA with the Kruskal-Wallis test followed by a Dunn’s multiple comparison test post hoc indicated as N.S. *P* > 0.05, *P < 0.05 and **P < 0.01.

During multivesicular body (MVB) formation, Shrub spiraled filaments drive membrane budding away from the cytosol, leading to formation of an intraluminal vesicle (ILV; Fig. 6A)^22, 34, 36, 41^. Shrub GOF can disrupt this process by overwhelming membrane trafficking with run-away filament formation^41^, due to an imbalance of ESCRT-III subunit stoichiometry. We hypothesized Shrub GOF would impair ILV formation, resulting in big luminal vesicles (BLV; Fig. 6A). Consistently, just such arrested trafficking organelles characterize both *dfmr1* null and *shrub* ^*OE*^ synaptic boutons (Fig. 6B). These aberrant compartments occur in the mutants only, and are not found in control boutons. In controls, ILVs are small, highly regular in size and never exceed 0.008 μm^2^ (Fig. 6B, top), whereas *dfmr1* and *shrub* ^*OE*^ BLVs are larger, display a greater variation in size and can be 10X larger at nearly 0.08 μm^2^ (Fig. 6B; middle and bottom). In quantified comparisons, BLV areas in *dfmr1* null (0.019 ± 0.007 μm^2^; *p* < 0.05) and *shrub* ^*OE*^ (0.02 ± 0.007 μm^2^; *p* < 0.01) PN boutons are both significantly larger than ILV areas in controls (0.007 ± 0.001 μm^2^; Fig. 6C). Importantly, BLVs are indistinguishable between FMRP LOF and Shrub GOF (*p* > 0.05; Fig. 6B,C). These results suggest elevated Shrub levels in the FXS disease state impair ILV formation and arrest membrane trafficking in synaptic boutons. The phenocopy of FMRP LOF and Shrub GOF defects is compelling, but to test causation we next corrected Shrub levels in the FXS condition and then assayed synaptic and membrane trafficking defects.

### Correcting Shrub levels in the FXS disease model mitigates synaptic errors

**Figure 7:**
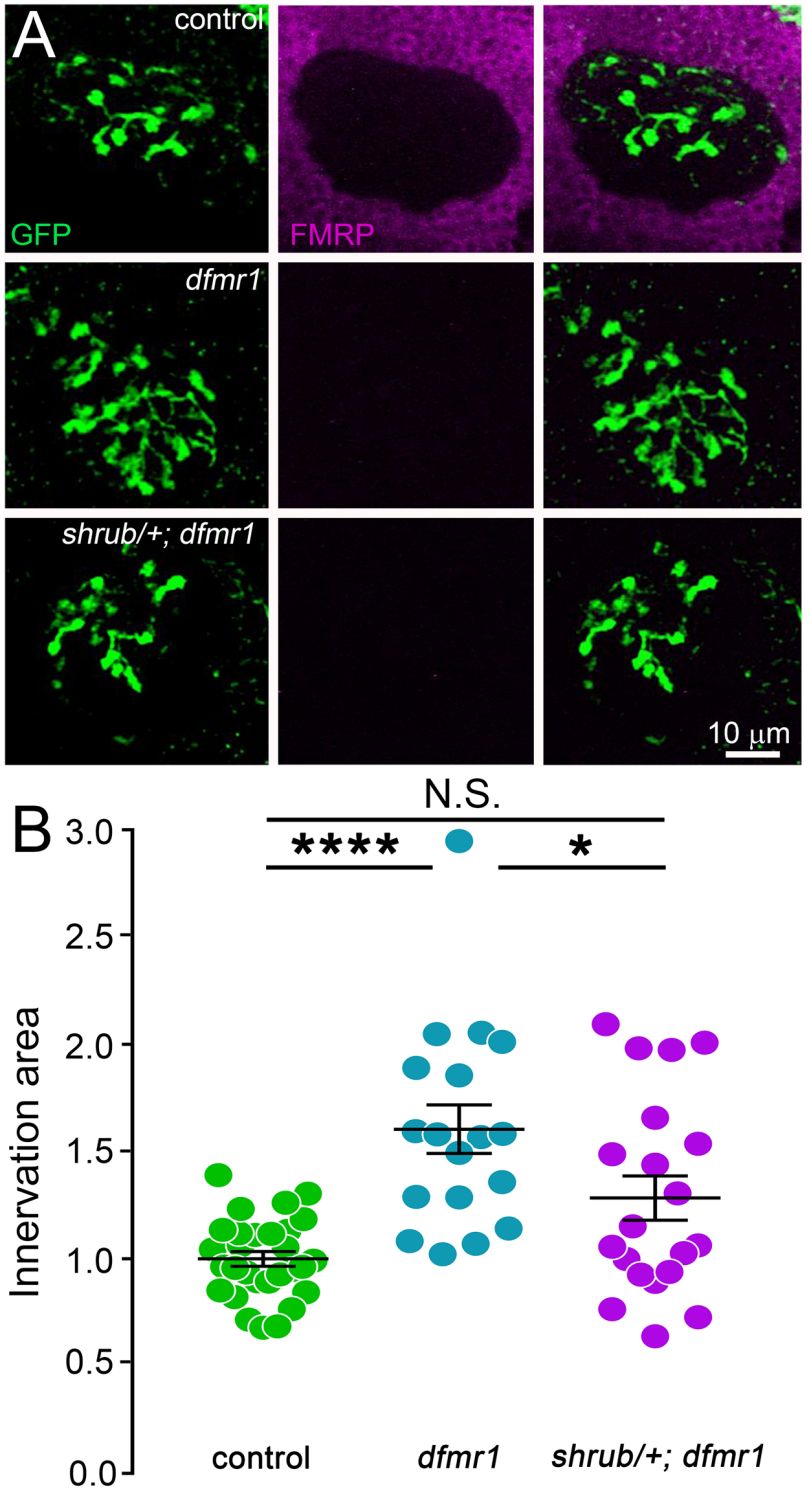
Shrub correction in *dfmr1* null mutants restores PN innervation of MB calyx. (**A**) Representative confocal images of PN innervation of the MB calyx comparing transgenic control (UAS-mCD8::GFP/+; Nrv3-Gal4/+), *dfmr1* null mutant (UAS-mCD8::GFP/+; Nrv3-Gal4, *dfmr1* ^*50M*^/*dfmr1* ^*50M*^) and *shrub* ^*4*^ null heterozygote (*shrub* ^*4*^/+) in the *dfmr1* homozygous null background (UAS-mCD8::GFP/*shrub* ^*4*^; Nrv3-Gal4, *dfmr1* ^*50M*^/*dfmr1* ^*50M*^). PN (green) in the calyx, with anti-FMRP labeled (magenta). (**B**) Quantification dot plot of MB innervation area showing mean ± SEM in the above three genotypes. Statistics done with one way ANOVA with the Kruskal-Wallis test followed by a Dunn’s multiple comparison test post hoc indicated as N.S. *P* > 0.05, *P < 0.05 and ****P < 0.0001.

**Figure 8:**
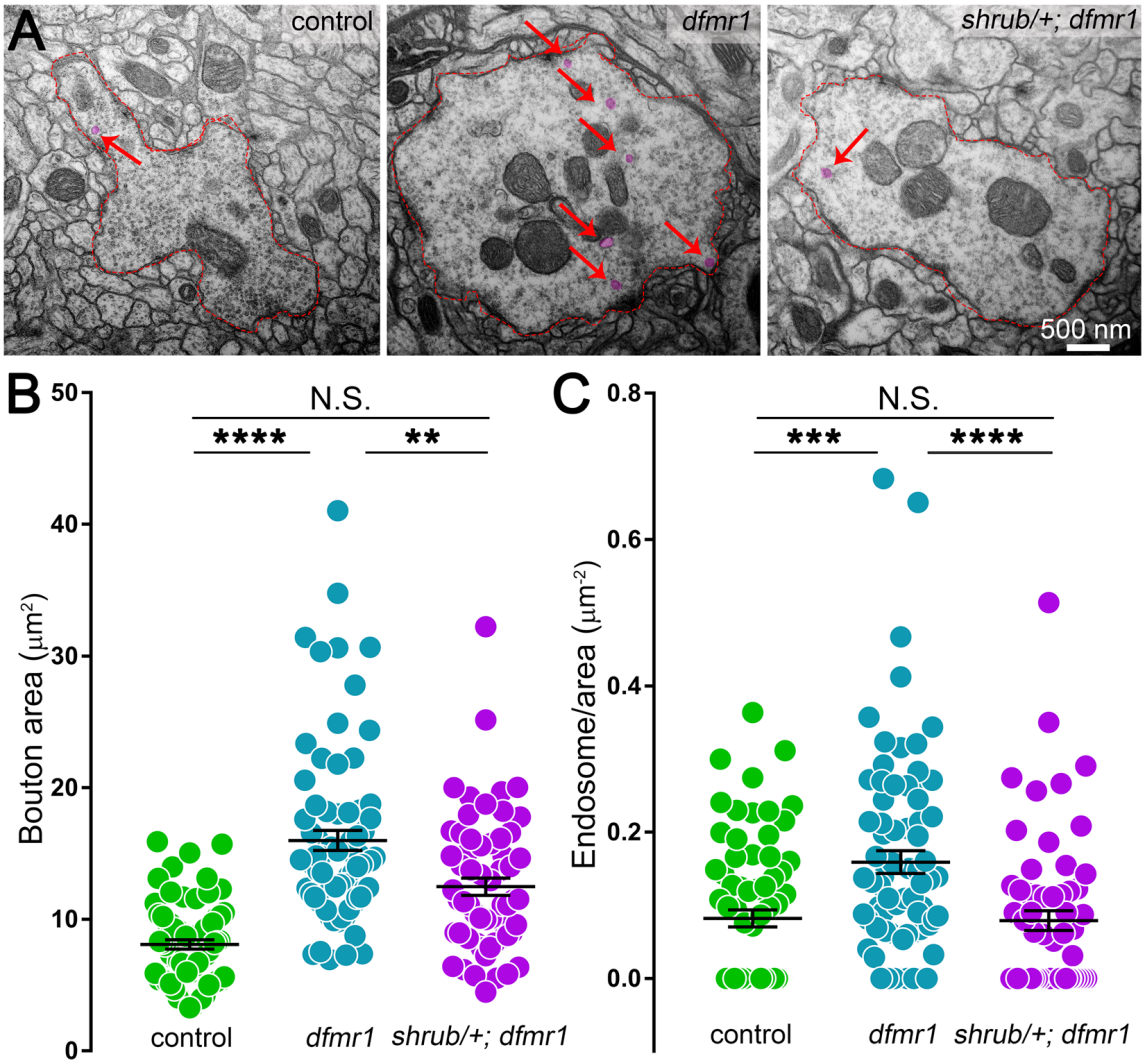
Shrub correction in *dfmr1* prevents PN synaptic bouton ultrastructure defects. (**A**) Representative electron micrographs comparing synaptic PN ultrastructure between the transgenic control (Nrv3-Gal4/+), *dfmr1* null mutant (Nrv3-Gal4, *dfmr1* ^*50M*^/*dfmr1* ^*50M*^) and *shrub* ^*4*^ null heterozygote (*shrub* ^*4*^/+) in *dfmr1* homozygous null background (*shrub* ^*4*^/+; Nrv3-Gal4, *dfmr1* ^*50M*^/*dfmr1* ^*50M*^). Dotted red lines depict PN synaptic bouton borders and red arrows indicate endosomic vacuoles, pseudo-colored (magenta). Quantification as dot plot for (**B**) bouton area and (**C**) endosome number per bouton area for the above genotypes. Statistics done with one way ANOVA with the Kruskal-Wallis test followed by a Dunn’s multiple comparison test post hoc indicated as N.S. *P* > 0.05, *P < 0.05, **P < 0.01, ***P < 0.001 and ****P < 0.0001.

The above results show FMRP LOF and Shrub GOF similarly cause overelaborated PN synaptic innervation of the central brain MB learning/memory center (Figs 2 and 4), as well as defective endosomal membrane trafficking within PN synaptic boutons (Figs 3, 5 and 6). We therefore hypothesized that FMRP-dependent Shrub suppression controls synaptic membrane trafficking to limit synaptic connectivity, with Shrub elevation due to FMRP loss causing the synaptic hyper-connectivity errors characterizing the FXS disease state. This hypothesis predicts that reducing Shrub levels towards normal in the *dfmr1* null mutant background should alleviate both FXS membrane trafficking defects and synaptic connectivity impairments. To test this hypothesis, we introduced a heterozygous *shrub* null allele (*shrub* ^*4*^ */*+)^24^ into *dfmr1* homozygous null animals. As previously, PNs plasma membranes were labeled with GFP (*shrub* ^*4*^ */*UAS-mCD8-GFP; *dfmr1* ^*50M*^, Nrv3-Gal4*/dfmr1* ^*50M*^) to assay phenotypes specifically within PN synaptic terminals. Under the same conditions, we compared the corrected *shrub* ^*4*^ */*+ heterozygous condition to both the genetic background (positive control) and FXS disease model (negative control). Representative confocal light microscopy images and quantified results for PN synaptic connectivity are shown in Fig. 7, and similar TEM data for synaptic membrane trafficking are shown in Fig. 8.

We first assayed PN innervation, again employing Nrv3-Gal4 > UAS-mCD8-GFP labeling immediately post-eclosion (3PE; Fig. 7A). Consistent with above results, transgenic controls (Nrv3-Gal4/+) display stereotyped innervation of the MB calyx, remaining constricted to a small subset of the total calycle region (Fig. 7A, top), and *dfmr1* null mutants exhibit obviously increased PN elaboration with spatially expanded innervation (Fig. 7A, middle). Consistent with our hypothesis, removing a single copy of *shrub* in the *dfmr1* null mutant background (*shrub* ^*4*^ */*+; *dfmr1/dfmr1*) is sufficient to strongly ameliorate the excessive PN innervation characterizing the FXS model (Fig. 7A, bottom). FMRP labeling in both *dfmr1* alone and with *shrub* ^*4*^ */*+ confirms the complete loss of FMRP in both genotypes compared to the control (Fig. 7A, middle column). With Shrub correction, PN innervation of MB calyx is more restricted in both area and spatial domain, more closely resembling the controls than *dfmr1* nulls (Fig. 7A). Consistent with these observations, removal of one copy of *shrub* in the *dfmr1* null background significantly reduces the PN innervation area within the MB calyx (1.3 ± 0.1; *p* < 0.05) compared to *dfmr1* alone (1.6 ± 0.1; *p* < 0.0001 vs. control), as normalized to control (Fig. 7B). Importantly, no significant difference (*p* > 0.05) remains between *shrub* ^*4*^ */*+; *dfmr1/dfmr1* and controls (1.0 ± 0.03; Fig. 7B). These results clearly demonstrate that increased Shrub levels strongly contribute to FXS synaptic connectivity errors. We next wanted to test roles of FMRP-dependent Shrub elevation in synaptic ultrastructure and membrane trafficking defects.

We assayed PN synaptic boutons using TEM immediately post-eclosion (3PE; Fig. 8A). Consistent with above results, transgenic control (Nrv3-Gal4/+) boutons are much smaller than *dfmr1* null boutons (left vs. middle). In contrast, Shrub correction reduces the bouton size (right). In quantitative comparisons, removing a single copy of *shrub* significantly reduces bouton area (12.48 ± 0.65 μm^2^, *n* = 62 boutons, *p* < 0.01) relative to *dfmr1* nulls (15.99 ± 0.76 μm^2^, *n* = 76; Fig. 8B). Importantly, no significant difference (*p* > 0.05) remains between *shrub* ^*4*^ */*+; *dfmr1/dfmr1* and controls (Fig. 8B). Finally, we tested whether correcting Shrub in the *dfmr1* background affects endocytic membrane trafficking at the synapse. Consistent with above results, control boutons contain low numbers of small endosomes, whereas boutons lacking FMRP show more, larger organelles (Fig. 8A, left and middle; pseudo-colored). Consistent with our hypothesis, correcting Shrub levels reduces the number of these compartments (right). When organelles per synaptic bouton area is quantified, both the controls (0.08 ± 0.01 μm^2^, *n* = 72 boutons, *p* < 0.001) and *shrub* ^*4*^ */*+; *dfmr1/dfmr1* (0.08 ± 0.0.1 μm^2^, *n* = 62, *p* < 0.0001) are significantly reduced compared to *dfmr1* alone (0.16 ± 0.02 μm^2^, *n* = 76; Fig. 8C). The control and Shrub correction conditions are identical, with no differences persisting (Fig. 8C). These results indicate the endocytic membrane trafficking errors in *dfmr1* null LOF synaptic boutons result from increased levels of Shrub. Collectively, the above findings strongly indicate that loss of FMRP-dependent Shrub regulation resulting in over-expression heavily impairs synaptic membrane trafficking to drive the synaptic hyper connectivity errors characterizing the FXS disease state.

## Discussion

Using a brain developmental proteomics screen in the *Drosophila* FXS model, we set out to find molecular players underlying synaptic connectivity errors^11–13, 63^. Here, we pursued a top candidate, the ESCRT-III protein Shrub (yeast Snf7, human CHMP4), a key component in select membrane trafficking mechanisms^22, 24, 41^. FMRP binds target transcripts to repress protein translation^8–11^, and we show here that FMRP binds *shrub* mRNA to limit Shrub protein levels specifically during the early-use critical period. This time window of initial sensory input closely following eclosion is delineated by peak FMRP expression levels and a transient requirement for FMRP in activity-dependent refinement mechanisms^4, 10^. FMRP mediates activity-dependent synaptic connectivity changes as an activity sensor regulating translation downstream of sensory input^2^. This FMRP function relies on phosphorylation, with sensory input activity driving dephosphorylation to remove translational inhibition^57^, and a FMRP phosphomimetic restoring protein levels and correcting synaptic connectivity errors in the *Drosophila* FXS model^15^. Using transient optogenetic stimulation immediately following eclosion, we show here that Shrub expression in neurons is acutely upregulated in an FMRP-dependent mechanism by activity during the early-use critical period, suggesting FMRP exerts translational repression of Shrub in this activity-dependent mechanism.

FMRP function in regulating synaptic connectivity is well established, with null mutants displaying increased complexity of both pre- and postsynaptic compartments^4, 12, 17, 63^. We show here that FMRP similarly limits olfactory projection neuron (PN) innervation of the mushroom body (MB) calyx, a key input into a central brain learning and memory center^47, 64^. Consistently, the ESCRT pathway also controls synaptic connectivity^24, 26–28^. Specifically, the ESCRT-III core component Shrub has critical roles shaping the architecture of *Drosophila* peripheral and central neurons. Loss-of-function (LOF) *shrub* mutations cause dendritic arbor overelaboration highly reminiscent of the *Drosophila* FXS disease model^24^. Critically, gain-of-function (GOF) in *shrub* homologs mimics LOF defects^41^, predicting that *shrub* GOF in the FXS state should have similar consequences. Consistently, we show here that PN-targeted *shrub* ^*OE*^ closely phenocopies *dfmr1* null defects of over-elaborated and over-extended innervation within the MB calyx. This parallel extends down to the level of individual PN synaptic boutons, which are similarly enlarged in both FMRP LOF and Shub GOF conditions, closely resembling the synaptic expansion characterizing other FXS model circuits^29, 61, 62^. These findings link Shrub function driving membrane trafficking to synaptic remodeling defects, strongly suggesting that elevated Shrub levels could be contributing to FXS connectivity errors.

Membrane from the cell surface of developing neurons is in a state of constant flux via endocytic budding and exocytic fusion, which requires tightly-regulated trafficking through the endomembrane organelle system^65^. The proper balance of membrane addition and retrieval is therefore essential for the surface remodeling that occurs during synaptic connectivity changes. For example, regulated endocytosis is required for dendritic arbor development of *Drosophila* sensory neurons, where endocytic membrane trafficking precedes synaptic reorganization^66^. Interfering with this membrane retrieval mechanism either through 1) blocking endocytosis (e.g. dynamin mutants) or 2) perturbing endosomic maturation (e.g. ESCRT pathway mutants) causes improper circuit wiring^25, 27, 28, 67^. Together with connectivity errors, the common membrane trafficking phenotype observed is the presence of irregular endocytic organelle compartments, visualized with endosomal markers such as Rab5^24, 28^. Specifically, mutations in ESCRT machinery such as Shrub result in the accumulation of enlarged endosomal organelles^24, 27^. We show here that consistent endomembrane trafficking defects with accumulated, greatly enlarged Rab5-positive endosomes occur in both *dfmr1* null LOF and *shrub* ^*OE*^ GOF synaptic boutons. These arrested endosomes are rarely observed in controls, suggesting that synaptic membrane trafficking errors due to Shrub over-expression characterize the FXS disease state.

Our synaptic ultrastructure studies consistently reveal accumulation of enlarged vacuoles in PN boutons, which are presumed to be arrested endosomes. Enlarged endosomes arise from impaired membrane trafficking flux, causing endosomes to swell as membrane accumulates^28, 41^. These findings suggest a bottleneck in synaptic membrane trafficking as a result of increased Shrub levels in the FXS condition, leading to an accumulation of arrested intermediates. We hypothesize this trafficking defect contributes to PN synapse errors in the FXS disease state. Indeed, *Drosophila* PN axons exhibit endosome clustering and fusion prior to MVB formation, with disruptions in this membrane traffic causing phenotypes similar to those reported here^67^. Our results support the conclusion that membrane trafficking is abnormal in the FXS disease state due to heightened Shrub levels. Although our results point to a endocytic membrane trafficking defect as causative, a study of ESCRT functions in synaptic remodeling suggest that while endosomal defects occur in all ESCRT pathway mutants, they may not be sufficient to explain synaptic errors^27^. This work revealed a second ESCRT module of Shrub acting locally at the plasma membrane to directly induce neural process fragmentation. We see no evidence of this mechanism in the MB calyx, but it is possible that this direct plasma membrane impairment contributes to synaptic defects in the FXS disease state.

In this study we focus attention on early adult brain PNs to demonstrate FMRP-mediated Shrub regulation modulates critical period synaptic connectivity. However, FMRP and Shrub act in other neuron types (e.g. sensory and motor neurons) and during various developmental periods (e.g. embryo and larva)^11, 24, 28, 68^. Thus, FMRP may regulate Shrub in multiple neuronal classes at different times. To date, no other published work links FMRP and Shrub. However, a recent study demonstrates FMRP regulates synaptic pruning of MB neurons (downstream of PNs) during metamorphosis^69^, a process where Shrub has a pivotal role^26–28^. Likewise, the well-studied neuromuscular junction (NMJ) model synapse shows A-D modulation^70^, with established roles for FMRP^11, 15^ and Shrub^24^ modulating connectivity during embryonic and larval development. These examples suggest possible overlaps in developing neurons, but what about mature neurons? Following the critical period when synaptic connectivity is refined, FMRP expression markedly decreases^10^, and some mature neurons do not display the overelaborated architecture characterizing *dfmr1* null mutants during development^4^. This implies a restricted role for FMRP at maturity, although Shrub is required for membrane traffic throughout life. Indeed, our results indicate a transient FMRP regulation of Shrub translation during critical period development. Thus, FMRP-Shrub interactions may be restricted to neuronal development stages.

Previous investigations linking membrane trafficking defects to synaptic connectivity errors focus on LOF mutants^26–28^, yet our findings show similar phenotypes from Shrub GOF. This duality may be explained by ESCRT-III subunit stoichiometry in assembled function. Unlike other ESCRTs, ESCRT-III components exist as auto-inhibited monomers that assemble sequentially on the membrane upon activation^41^, with the relative abundance of ESCRT-III subunits paramount to membrane trafficking functions. Indeed, yeast studies demonstrate that increased levels of the Shrub homolog Snf7 strongly hamper ESCRT-III function, resulting in endosomal trafficking defects similar to LOF mutants^41^. The rationale is that Shrub/Snf7 continues to form protein filaments unless capped by Vps24, a critical checkpoint step that allows membrane trafficking to proceed forward^41^. When Shrub/Snf7 is in stoichiometric excess, capping does not occur properly and filaments fail to stop polymerizing, resulting in impaired membrane trafficking. We show here that aberrantly enlarged intraluminal vesicle organelles (BLV compartments) occur only in FMRP LOF and Shrub GOF synaptic boutons, consistent with unrestrained Shrub filament formation at trafficking endosomes. Given arrested BLV compartments characterize both *dfmr1* null and *shrub* ^*OE*^ boutons, we propose a membrane trafficking impairment underlies synaptic connectivity defects in the FXS disease state.

Our work suggests a cell-autonomous role for FMRP in suppressing Shrub translation to regulate synaptic refinement within the neuron. However, we do not preclude the possibility of intercellular signaling and, indeed, FMRP is well known to regulate intercellular signaling in the *Drosophila* FXS model^16, 29^. Moreover, ESCRT-III membrane trafficking is also well known to regulate the presentation of cell surface proteins involved in intercellular signaling^26^. In this context, glia are known to sculpt connectivity during development, with glial phagocytosis synaptic pruning conserved from insects to mammals^71–74^. A recent *Drosophila* study demonstrated that FMRP loss causes reduced glial phagocytosis during metamorphic neuronal pruning^69^. It is possible that a similar mechanism occurs during the later, activity-dependent refinement. This mechanism could involve two steps; 1) Shrub-dependent, cell-autonomous synapse marking, and 2) consequent pruning via glial engulfment. Studies suggest Shrub-dependent processes act upstream of glial synapse elimination, and may be required for glial activation^26–28^. Although beyond the scope of the current study, it is interesting to speculate that elevated Shrub levels in the FXS condition could disrupt cell-autonomous synapse tagging, preventing glial engulfment. Future studies will probe connections between Shrub membrane trafficking and glia in the FXS disease state.

We demonstrate here that lack of FMRP-mediated Shrub regulation strongly contributes to FXS synaptic defects. However, we note that Shrub is only a piece of the puzzle. FMRP regulates many neural transcripts^56^, although the scope of this regulation remains indeterminate, and a number of these targets are established to play roles in synaptic connectivity. Validated targets like Microtubule Associated Protein 1B (*Drosophila* Futsch) control the cytoskeleton during synaptic outgrowth^11^, while others such as matrix metalloproteinases (MMPs) modulate *trans*-synaptic signaling^29^. Whether these targets act in the same, parallel or distinct pathways in the FXS context is entirely unknown. However, it is interesting to envision a coupled mechanism in which they work together to manifest disease impairments. For example, extracellular MMPs could control information relay at synapses via surface receptors that are impacted by Shrub-mediated membrane trafficking supported by cytoskeletal changes regulated by Futsch. Sculpting synapses could thereby require appropriate extracellular, membrane and cytoskeletal remodeling in a linked cascade. This linkage could potentially explain why manipulating individual FMRP targets can rescue FXS synaptic connectivity phenotypes. Investigations involving modulating multiple targets in combination in the disease model background will be required to dissect the complex interactions between FMRP targets.

This study reveals for the first time a link between ESCRT-III membrane trafficking and FXS synaptic connectivity defects. Correcting Shrub levels prevents synaptic membrane trafficking defects and mitigates synaptic connectivity errors. Our results suggest FMRP directly negatively regulates Shrub translation to control Shrub-dependent synaptic membrane traffic driving synaptic refinement. ESCRT-III has been similarly implicated in other neurological disorders. For example, mutations in the human ESCRT-III component CHMP2B are linked to frontotemporal dementia (FTD), with defects in endocytic membrane trafficking similar to those reported here^48^. However, FTD causes neurodegeneration, seemingly at the opposite end of the neurological spectrum from synaptic overgrowth. However, our study and others have focused on neurodevelopment, while FTD occurs in aging neurons. This raises the interesting possibility that disrupting the same membrane trafficking pathway could lead to opposite effects depending on age, underscoring the importance of developmental timing in studying neurological disorders. Future studies are required to dissect how Shrub-dependent membrane trafficking errors underlie synaptic connectivity defects in the FXS disease state. However, our current results clearly implicate increased Shrub levels in innervation abnormalities in the *Drosophila* FXS model brain learning and memory center, promising novel avenues for FXS therapeutic intervention.

## Methods

### Drosophila genetics

Stocks were reared on standard cornmeal/agar/molasses food at 25 °C on a 12 h light:dark cycle. All lines were generated with standard genetic crosses schemes with the following starter stocks: (1) *w* ^1118^ genetic background control, 2) *dfmr1* ^*50M*^/TM6TbGFP null allele^11^, 3) UAS-9557–3 *dfmr1* transgene^11^, 4) *shrub* ^*4*^/Cyo null allele^24^, 5) UAS-*shrub* transgene^24^, 6) *elav*-Gal4/Cyo pan-neuronal driver (Bloomington stock # 8765), 7) Nrv3-Gal4 selective projection neuron driver (Janelia FlyLight Collection R54F05)^60^, 8) UAS-ChR2(H134R)-mCherry channelrhodopsin transgene^4^, and 9) UAS-mCD8-GFP membrane marker^4^. *w* ^1118^ was used as genetic background control. Transgenic controls used were *elav*-Gal4/+ and Nrv3-Gal4/+. All genotypes were run in parallel for each experimental replicate.

### Western blots

The brains of staged animals were dissected on ice in phosphate buffered saline (PBS) containing 1X Roche complete EDTA free protease inhibitor (Roche 04 693 123 001) and immediately snap-frozen on dry ice. Samples were homogenized in lysate buffer containing 1X LDS (NuPAGE LDS Sample Buffer 4X Life Technologies NP0007), 1X ΣPI, 1X PMSF and 1.3% BME in ddH_2_0, and then incubated at 4 °C for 30 mins with rotation. Lysates were spun down at 16000 g for 10 mins at 4 °C, and supernatant was then heated for 10 mins at 70 °C followed by a 10 min 16000 g centrifugation. Lysate from 4 brains was loaded into 4–12% Bis-Tris gels with MES running buffer, and run for 10 mins at 100 V followed by a shift to 150 V until the dye ran off. Samples were transferred to nitrocellulose membranes overnight (O/N) at 4 °C using 1X NuPAGE transfer buffer (novex NP0006-1), supplemented with 20% methanol (MeOH). Membranes were rinsed with ddH_2_0 and then blocked in 5% powdered milk in Tris buffered saline (TBS: 150 mM NaCL, 5 Mm KCl, 25 mM Tris) for 2 h at room temperature (RT) with rotation. Membranes were incubated in primary antibody diluted in 5% powdered milk in TBS with 0.1% Tween-20 (TBS-T) O/N at 4 °C with rotation. Blots were then washed 6 × 5 mins in 5% powdered milk in TBS-T at RT with rotation followed by a 2 h incubation with secondary antibody diluted in wash buffer. Membranes were washed as above then imaged using a Li-Cor Odyssey machine. Protein bands were analyzed using Image Studio software normalized to loading controls, then presented as percent of control. Primary antibodies used were: rabbit anti-Shrub (1:10000), a gift from Fen Biao Gao^24^, mouse anti-dFMRP (1:3000) (Sigma F4554) and mouse anti-α-tubulin (1:5000) (DHSB 12G10). Secondary antibodies used were: Alexaflour 680 goat anti-rabbit and -mouse (1:10000).

### RNA immunoprecipitation

RNA immunoprecipitation (RIP) was done as previously described^11, 45^, with minor changes. Staged animals were decapitated and the heads snap-frozen on dry ice in nuclease free tubes. 100 heads per genotype were homogenized with a pestle in 600 µl lysis buffer (20 mM hepes, 100 mM NaCl, 2.5 mM EDTA, 0.05% Triton X, 5% glycerol, 1X SigmaFast Protease inhibitor, 120 U/ml RNAse inhibitor). The lysate was incubated for 30 mins at 4 °C with rotation, and then spun at 12,000 g for 10 mins at 4 °C. The supernatant was transferred to prechilled tubes containing non-conjugated DynaBeads (Invitrogen 10003D), and allowed to preclear for 30 mins at 4 °C with rotation. DynaBeads were conjugated to monoclonal mouse anti-dFMRP (Sigma F4554) according to the manufacturer’s protocol, and precleared lysate (200 µl) was added to the antibody-bead slurry (80 µl) O/N at 4 °C with rotation. The beads were then separated from supernatant and washed 3X with 500 µl fresh lysis buffer vortexing with 500 µl TRIzole (ambion 15596026). Chloroform (100 µl) was added and samples were vortexed for 30 sesc and then incubated for 2 mins. Samples were then centrifuged at 16000 g for 10 mins at 4 °C. The upper aqueous layer was transferred to prechilled tubes, and glycogen (1 µl) then 2-propanol (250 µl) added and incubated for 10 mins at RT with rotation. Samples were then spun at 16000 g for 10 mins at 4 °C, the supernatant removed and the pellet washed 3X with 75% EtOH (500 µl), followed by centrifugation at 16000 g for 10 mins at 4 °C. EtOH was then removed and pellets air-dried for 10 mins. ddH_2_0 (20 µl) was added and mRNA reverse transcribed with a SuperScript Vilo kit (Invitrogen 11754-050) according to manufacturer’s protocol. cDNA was amplified with HotSart Taq (New England BioLabs M0495S) using a touchdown PCR protocol. Amplified cDNA was separated on a 0.8% agarose gel and visualized with SyberSafe DNA stain (Invitrogen S33102). Gels were run at 150 V for 1 h. PCR primers used: *shrub* forward primer GTTTCTTCGGGAAGATGTTCGG, *shrub* reverse primer CTGATGGGCTCTCTTGAGGG, *futsch/map1b* forward primer CAGTTTCACCCGCCACCG, *futsch/map1b* reverse primer GCACGTTGCTGTTGTTTAGGC, *α*-*tubulin* forward primer CTGTGGTCGATGAGGTCCG *α*-*tubulin* reverse primer GCGTAGGTCACCAGAGGG.

### Optogenetic stimulation

Optogenetic experiments were performed as previously described^4^. Briefly, staged animals expressing channelrhodopsin (UAS-ChR2(H134R)-mCherry) under the *elav*-Gal4 pan-neuronal driver were fed from hatching on standard food supplemented with either 10 μl EtOH vehicle (in 10 ml volume; control) or with 10 μM all-trans retinol (ATR), an essential cofactor^4^. Immediately following eclosion, animals were placed in 30 mm petri dishes with pleated Whatman paper saturated with vehicle control or ATR in a 20% sucrose solution. Sealed petri dishes were placed in a LED exposure chamber with two Luxeon Rebel Endor Star 3 × 15-Watt LED arrays (470 nm blue light, LED Supply). At 15 V, the LED arrays generate ~100 μW/cm^2^ at a working distance of 2 cm. Animals were exposed to 5 Hz pulses of blue light for 2 h and subsequently processed for Western blotting as described above.

### Immunocytochemistry imaging

Staged animal brains were labeled and imaged as previously reported^5^, with slight variation. Briefly, brains were dissected in cold PBS and immediately fixed using 4% paraformaldehyde (PFA) + 4% sucrose + Triton-X in PBS (PBST) for 30 mins at RT with rotation. Samples were then rinsed 3X with PBS and blocked for 2 h in 1% bovine serum albumin (BSA) + 0.5% normal goat serum (NGS) or without NGS if depending on secondary used in PBST at RT with rotation. Samples were incubated in primary antibody at 4 °C O/N with rotation, washed 3 × 20 mins in PBST at RT with rotation, and then incubated with secondary antibody for 4 h at RT with rotation. Antibodies were diluted in 0.2% BSA with or without 0.1% NGS depending on secondary in PBST. Primary antibodies used were: rabbit anti-GFP (1:2000) (Abcam 290), FitC conjugated goat anti-GFP (1:500) (Abcam 6662), rat anti-mCD8 (1:100) (eBioscience), mouse anti-Brp (1:100) (DHSB nc82), rabbit anti-Shrub (1:1000)^24^, and rabbit anti-Rab5 (1:200), a gift from Fengwei Yu^28^. Secondary antibodies used were: AlexaFluor 568 goat anti-mouse, AlexaFluor 488 goat anti-rabbit and -rat, AlexaFluor 488 donkey anti-rat and -goat, and AlexaFluor 568 donkey anti-rabbit (all 1:250). Labeled brains were mounted in Flouromount (EMS 17984-25) with posterior up. Images were acquired on a Zeiss LSM 510 Meta confocal microscope using Plan Neofluar 40X oil immersion objectives with a numerical aperture of 1.3.

### Electron microscopy

Electron microscopy was done as previously reported^17^, with slight variations. Dissected brains from staged animals were immediately fixed in 2.5% gluteraldehyde (GTA) in 0.1 M sodium cacodylate (SC) buffer (pH 7.4) O/N at 4 °C. Samples were then washed 3 × 20 mins with 0.1 M SC buffer at RT followed by a final wash in 0.1 M SC buffer O/N at 4 °C. Samples were fixed with 1% osmium tetroxide in 0.1 M SC buffer in glass tubes for 1 hr at RT, and then washed 3 × 20 mins with 0.1 m SC buffer. Samples were treated with 2% uranyl acetate (UA) in ddH_2_0 for 2 hr at RT covered, followed by 3 × 20 min washes in ddH_2_0. Samples were then placed through an EtOH dehydration series (30, 50, 70 90, 95, 2 × 100%) for 10 min each at RT. Propylene oxide (PO) was used as a transitional solvent: 50/50 EtOH/PO, 2 × 100 PO for 10 min each, 75/50 PO/Epon-812 resin for 30 mins, 50/50 PO/Epon-812 for 1 hr, and 50/50 PO/Epon-812 O/N at RT. Fresh Epon-812 was then allowed to infiltrate tissue for 2 hr, 4 hr and O/N at RT. Flat block molds were half filled with Embed-812 resin and heated at 60 °C for 4 hr or until resin reached a tacky consistency. Samples were placed in blocks oriented anterior up and dorsal forward. Blocks were filled with resin and polymerized for 48 hr at 60 °C. Thick sections (500 nm) were made using a Leica Ultracut UCT microtome until the dorsal most region of the brain was reached. Thick sections were continued for another 40 μm. Thin sections (65 nm) were then taken and collected on formvar-coated slotted grids (Electron Microscopy Sciences) at 10 sections per grid. PN boutons were chosen at random from within each MB calyx. Only 1 section per grid was imaged, with at least 5 grids (~3 μm) between each grid examined. Images were acquired using a Philips CM10 transmission electron microscopy at 80 kV.

### Analysis statistics

All measurements were done using ImageJ^75^. For area measurements (Figs 2B,C and 7) images were processed with the despeckle tool and threshold signals then selected using the wand tool in ImageJ. Rab5 studies (Fig. 3) were performed on single optical slices, with boutons selected at random and outlined with the free hand tool in ImageJ. All genotypes were run in parallel at the same time and under the same conditions for each replicate. All statistical analysis was performed using Prism (GraphPad Software, San Diego, CA). Data from two group comparisons were analyzed with a two-tailed unpaired t-test. Data from three or more comparisons were analyzed with one-way analysis of variance (ANOVA) with the Kruskal-Wallis test followed by a Dunn’s multiple comparison test, comparing each mean to every other column. In figures, significant levels are shown as p > 0.05 (not significant; N.S.), *p < 0.05, **p < 0.01, ***p < 0.001 and ****p < 0.0001.

### Data availability

All datasets generated and analyzed for the current study are available upon reasonable request from the corresponding author.

## Electronic supplementary material

Supplemental Figure S1
