# Supplementary material for: ESCRT-III Membrane Trafficking Misregulation Contributes To Fragile X Syndrome Synaptic Defects
Source: Sci Rep. 2017 Aug 17;7:8683. doi: 10.1038/s41598-017-09103-6 (PMC5561180; doi:10.1038/s41598-017-09103-6)
Supplement: Supplementary file 1 — Supplemental Figure S1 [file 41598_2017_9103_MOESM1_ESM.pdf]

# ESCRT-III Membrane Trafficking Misregulation Contributes To Fragile X Syndrome Synaptic Defects Supplementary Information

**Dominic J. Vita[1] and Kendal Broadie[1,2,3,*]**

[1]Vanderbilt University, Department of Biological Sciences, Nashville, Tennessee, 37235, USA
[2]Vanderbilt University, Kennedy Center for Research on Human Development, Nashville, Tennessee 37235, USA
[3] Vanderbilt University, Vanderbilt Brain Institute, Nashville, Tennessee, 37235, USA

*Corresponding author:
Kendal Broadie
1210 MRBIII
VU Station B, Box 35-1634
Nashville, TN 37235 USA
Tel: 615-936-3937
kendal.broadie@vanderbilt.edu

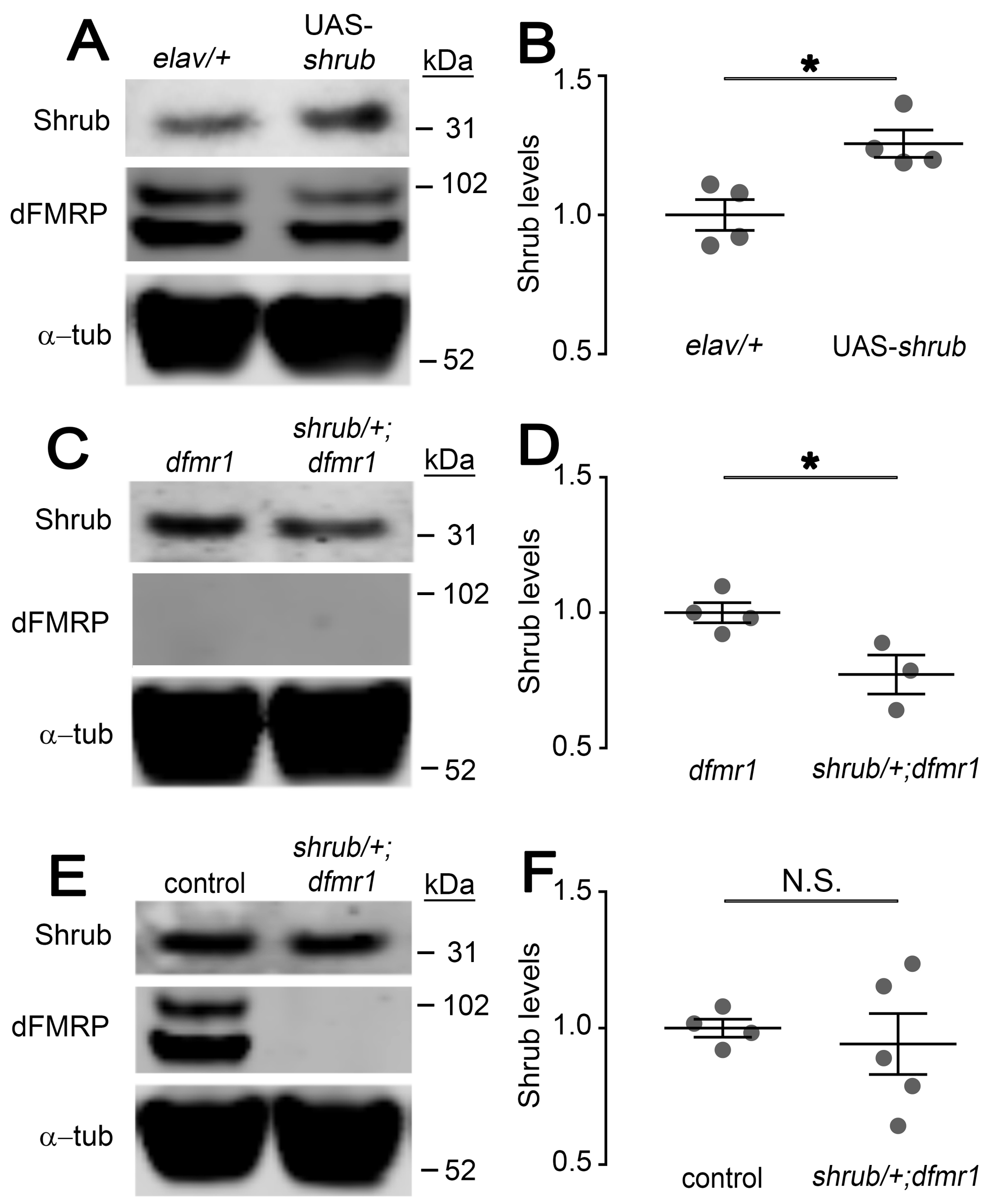


**Supplemental Figure S1: Brain Shrub levels with *shrub* genetic manipulations**
**A)** Brain Western blot comparing *elav*-Gal4/+ driver control to *elav*-Gal4/+>UAS-*shrub* at 0-3hr post-eclosion (3PE). Proteins probed are indicated on left, molecular weights on right. **B)** Quantification dot plots showing all data points, with mean±SEM. **C)** Brain Western blot comparing the *dfmr1* null mutant to $shrub^{4}$/+ null heterozygote in the *dfmr1* background ($shrub^{4}$/+; $dfmr1^{50M}$/$dfmr1^{50M}$) at 3PE. Proteins probed are indicated on left, molecular weights on right. **D)** Quantification dot plots of data points, with mean±SEM. **E)** Brain Western blot comparing control (*Nrv3*-Gal4/+) to *shrub*/+; *dfmr1* ($shrub^{4}$/+; *Nrv3*-Gal4, $dfmr1^{50M}$/$dfmr1^{50M}$) at 3PE. Proteins probed are indicated on left, molecular weights on right. **F)** Quantification dot plots of data points, with mean±SEM. Statistics were done with two tailed unpaired t-test, indicated as $P<0.05$ (*) and $P>0.05$ (N.S.).
